# Physical exercise for a healthy pregnancy: the role of placentokines and exerkines

**DOI:** 10.1186/s12576-023-00885-1

**Published:** 2023-11-14

**Authors:** Hamed Alizadeh Pahlavani, Ismail Laher, Katja Weiss, Beat Knechtle, Hassane Zouhal

**Affiliations:** 1 Department of Physical Education, Farhangian University, P.O. Box 14665-889, Tehran, Iran; 2 Department of Anesthesiology, Pharmacology, and Therapeutics, Faculty of Medicine, University of British Columbia, Vancouver, BC Canada; 3 Institute of Primary Care, University of Zurich, Zurich, Switzerland; 4 Medbase St Gallen Am Vadianplatz, Vadianstrasse 26, 9001 St. Gallen, Switzerland; 5 Movement Sport, Health and Sciences Laboratory (M2S) UFR-STAPS, University of Rennes 2-ENS Cachan, Charles Tillon, France; 6 Institut International Des Sciences Du Sport (2IS), Irodouer, France

**Keywords:** Exercise, Pregnancy, Placentokine, Exerkine, Diabetes, Preeclampsia

## Abstract

Complications such as diabetes and preeclampsia can occur during pregnancy. Moderate-intensity exercise can prevent such complications by releasing placentokines and exerkines, such as apelin, adiponectin, leptin, irisin, and chemerin. Exercise and apelin increase thermogenesis and glucose uptake in pregnancy by activating AMPK, PI3K, PGC-1α, AKT1, UCP3, and sarcolipin. Exercise increases apelin levels to reduce preeclampsia symptoms by increasing eNOS, NO, placental growth factor (PlGF), and VEGF and decreasing levels of fms-like tyrosine kinase 1 (sFlt-1), soluble endoglin (sEng), and oxidative stress. A negative relationship has been reported between plasma leptin and VO_2_peak/kg and VO_2_peak in women with gestational diabetes. In active women, decreases in leptin levels reduce the risk of preeclampsia by ~ 40%. Higher adiponectin levels are associated with greater physical activity and lead to increased insulin sensitivity. Increased adiponectin levels in preeclampsia and exercise counteract inflammatory and atherogenic activities while also having vascular protective effects. Exercise increases irisin levels that correlate negatively with fasting glucose, insulin concentration, and glycosylated hemoglobin levels. Irisin augments mRNA expression levels of UCP1 and cell death-inducing DNA fragmentation factor-like effector A (cidea) to cause browning of adipose tissue, increased thermogenesis, and increased energy consumption. Irisin concentrations in mothers with preeclampsia in the third trimester negatively correlate with systolic and diastolic blood pressure. Expression levels of chemerin, IL-6, and TNF-α are increased in gestational diabetes, and the increases in chemerin in late pregnancy positively correlate with the ratio of sFlt-1 to PlGF as a marker of preeclampsia. The effects of physical exercise on placentokines and exerkines in women at various stages of pregnancy remain poorly understood.

## Introduction

Both physiological and pathological changes occur during pregnancy to nurture the fetus and prepare the mother for delivery of the baby [1]. Pregnancy affects the metabolism of the mother and the fetus, with 2–18% of pregnant women developing gestational diabetes that can cause fetal loss, stillbirth, premature delivery, hypertrophic cardiomyopathy, respiratory distress syndrome, neonatal hypoglycemia, accelerated fetal growth, and macrosomia [2]. Maternal complications in gestational diabetes include preeclampsia, increased liver enzymes, low platelet syndrome, hypoglycemia, and kidney failure [2, 3]. Insulin resistance at 22–26 weeks of gestation is an independent predictor of preeclampsia [4]. The risk of preeclampsia in women with type 1 or 2 diabetes increases by ~ 2 to 4 times [4, 5]. Preeclampsia is defined as the occurrence of high blood pressure in a healthy woman at or after the 20th week of pregnancy and is associated with maternal mortality (approximately 50,000 deaths/year worldwide) [6]. Complications of preeclampsia after pregnancy include chronic hypertension, diabetes, ischemic heart disease, cerebrovascular disease, kidney disease, thromboembolism, hypothyroidism, and even memory impairment [7].

The American College of Obstetricians and Gynecologists recommends moderate exercise (30 min or more per day) for pregnant women on most days of the week [8]. Increasing energy consumption to at least 16 METs per week or preferably 28 METs per week and increasing exercise intensity to 60% of the reserve heart rate during pregnancy reduces the risk of gestational diabetes and preeclampsia [8]. Exercise during pregnancy improves the health of the mother and fetus and has long-term benefits for the mother [9]. For example, women who exercise (3 times a week, for at least 20 min per session) in early pregnancy report less discomfort in late pregnancy [10], and women who are physically active during pregnancy experience a 40–70% reduced risk of developing gestational diabetes [11]. Recreational physical activity during pregnancy is associated with a reduced risk of preeclampsia [12]. Thus, exercise during pregnancy can reduce the risk of developing preeclampsia and gestational diabetes [13, 14].

The benefits of exercise in pregnancy likely occur through the release of placentokines (cytokines released from the placenta) and exerkines (cytokines released during exercise), both of which affect the physiology of the body during pregnancy and exercise. For example, physical activity during pregnancy improves oxidative metabolism, fetal growth, and maternal metabolism through the release of exerkines and placentokines, which increases mitochondrial biogenesis and PGC-1α expression, leading to muscle growth and increased brown fat deposition in the muscle of the offspring [15]. On the other hand, circulating irisin concentrations increase during human pregnancy and regulate placental trophoblast cell differentiation by activating AMPK, while circulating levels of irisin are reduced in preeclampsia and gestational diabetes [16]. This review summarizes the roles and mechanisms of action of exerkines and placentokines in gestational diabetes and preeclampsia and discusses the role of exercise in improving gestational diabetes and preeclampsia.

### Exerkines and placentokines

Tissues secrete a variety of cytokines, such as myokines (secreted by muscle), adipokines (secreted by adipose tissue), and placentokines (secreted by the placenta), while myokines, adipokines, and placentokines that are released by exercise can also be referred to as exerkines. For example, exercise increases the release of irisin, which is both a myokine and an adipokine [15] (Table 1).

**Table 1:** Placentokines and exerkines in pregnancy

Placentokines & exerkines	Action	References
Apelin	Increased metabolism, thermogenesis in humans and animals	[17]
	Increased glucose transport, decreased insulin resistance in animals	[21–24]
	Increased lipid oxidation, increased brown adipose tissue in animals	[20]
	Increased mitochondrial activity, mitochondrial biogenesis, increased type 1 fibers in animals	[20]
	Reduce oxidative stress, decreased preeclampsia in animals	[27]
	Reduced blood pressure, increased heart rate, increased ejection fraction, and baroreflex sensitivity in animals	[28]
	Vasodilator effect, nitric oxide (NO) production, smooth muscle relaxation in animals	[29]
	Increases in angiogenesis in humans and animals	[30, 31]
Leptin	Inhibition of insulin secretion in humans	[32]
	Increased muscle mass and muscle fiber size, decreased expression of myostatin, muscle loop protein 1 (MuRF1), and muscle atrophy F-box (MAFbx) in humans and animals	[33]
	Increased hydrolysis and oxidation of fatty acids, and muscle triglycerides (TG) in humans and animals	[34, 35]
	Production of TNF-α, IL-6, and IL-12 by monocytes, and increased diabetes in humans and animals	[35]
	Increased sFlt-1/PlGF ratio, increased preeclampsia in humans	[36]
	Increased anti-angiogenic factors, such as sFLT1, s-endoglin, endothelin 1 in humans	[37]
	Thrombus formation and increased atherosclerosis due to proliferation, migration, and calcification of vascular smooth muscle cells (VSMCs) in animals	[38]
	Increased blood pressure, heart rate, pathological hypertrophy, left ventricular dysfunction, frequency of ischemic arrhythmias, systemic inflammation, and myocardial infarction size in animals	[39]
Adiponectin	Glucose and lipid metabolism, anti-inflammatory effect in humans	[40]
	Increased insulin sensitivity, increased fatty acid oxidation and glucose uptake in skeletal muscles, decreased glucose production in the liver in humans and animals	[41]
	Has anti-inflammatory, anti-atherogenic effects and vasoprotective activities in humans	[42, 43]
	Decreased cell adhesion molecules [E-selectin, intercellular adhesion molecule (ICAM-1), vascular cell adhesion molecule (VCAM-1)] and reduced vascular inflammation in humans	[44]
Irisin	Decreased insulin resistance in humans	[111, 45]
	Antioxidant properties in humans and animals	[46]
	Increased PGC-1α, nuclear respiratory factor 1 (NRF1), and mitochondrial transcription factor A (TFAM), increased mitochondrial content and oxygen consumption in animals	[47, 48]
	Increased mRNA expression of UCP1 and Cidea, browning of subcutaneous and visceral adipose tissue, and thermogenesis in humans and animals	[49]
	Anti-apoptotic, decreased caspase-3 activity, increased anti-apoptotic BCL2 to pro-apoptotic BAX, decreased ROS, increased Akt signaling pathway and cell survival in humans	[50]
Chemerin	Decreased tissue glucose uptake and increased insulin resistance in human muscle cells in humans	[51]
	Increased fatty acid binding protein 4 (FABP4), and inflammatory factors IL-6 and TNF-α in humans	[52]
	Increased preeclampsia positively correlated with the ratio of soluble Fms-like tyrosine kinase-1 (sFlt-1) to placental growth factor (PlGF) in humans	[53]
	Increased sFlt-1, inflammatory markers (NFkB, TNF-α, and IL-1β), reduced vascular endothelial factor A (VEGF-A), and inhibition of tube formation during co-cultured with human umbilical vein endothelial cells (HUVECs) in animals	[54]

### Apelin as a placentokine and exerkine in gestational diabetes

**Fig. 1:**
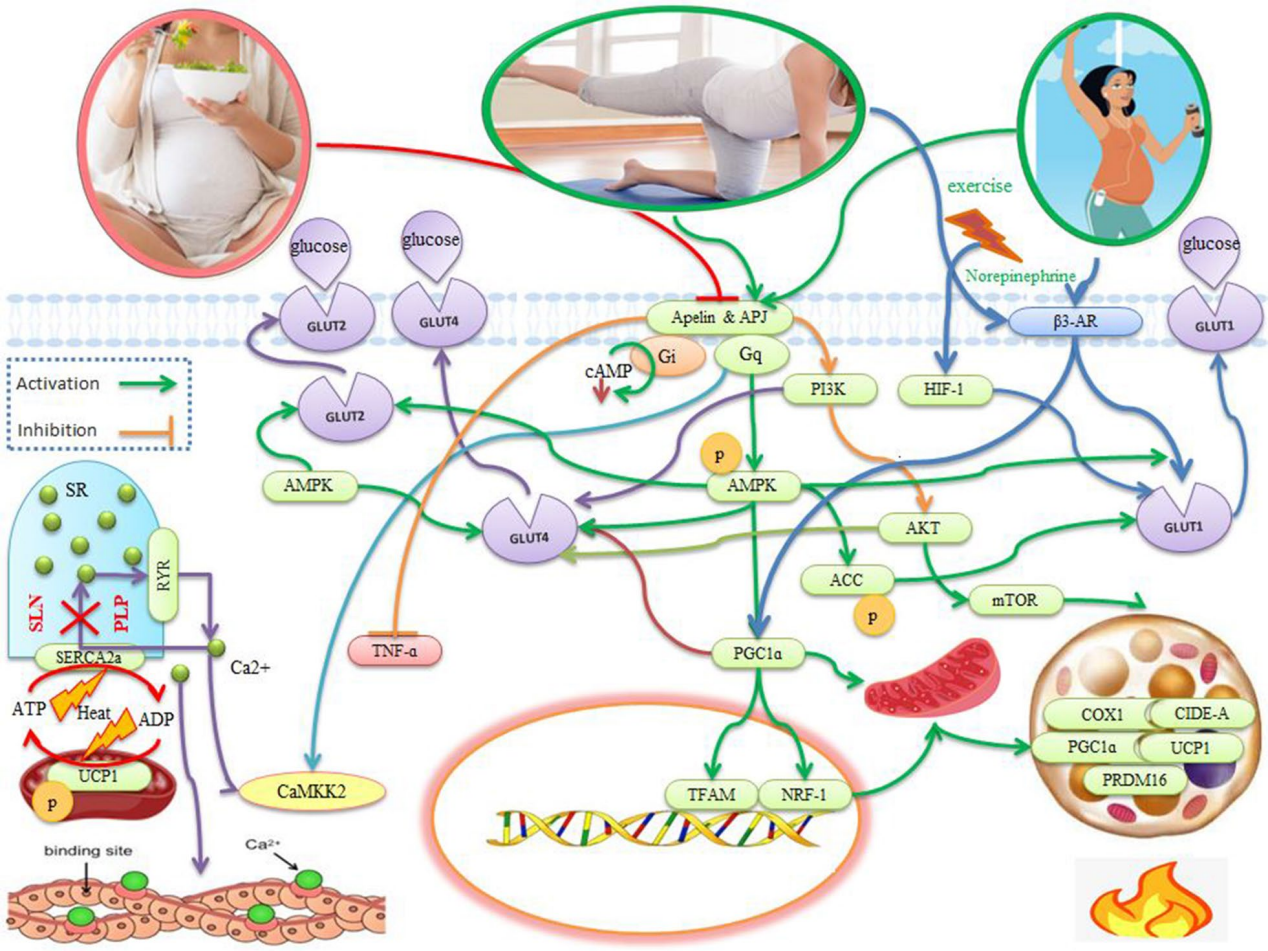
Effects of apelin (as an exerkine and placentokine) on gestational diabetes in muscles and adipocytes. Apelin couples to the apelin receptor (APJ) and via Gαq activates CaMKK2 to stimulate thermogenesis. In myocytes, SERCA in the sarcoplasmic reticulum (SR) membrane transports calcium from the cytosol to the SR using ATP hydrolysis. Under hyperthermia conditions, SERCA activity is inhibited by phospholamban (PLP) or sarcolipin (SLN), but its ATPase activity remains. Therefore, to transport Ca2 + into the SR, mitochondrial ATP synthesis is increased and heat is generated. Apelin significantly activates AMPK via Gαq, then increases glucose transport by stimulating GLUT4 and GLUT1 in the heart and skeletal muscle. By activating PGC-1, exercise training stimulates mitochondrial biogenesis, increases slow-twitch fibers, and increases glucose uptake through GLUT4. Maternal exercise activates AMPK and PGC-1α in fetal muscle and then increases the expression of UCP3 and sarcolipin. This process inhibits the uptake of calcium ions into the sarcoplasmic reticulum, thereby activating CaMKK2 to generate heat through muscle contraction. After exercise, increased PI3k and AKT1 also increase GLUT4 stimulation. In addition, exercise-induced hypoxia may cause glucose transport through GLUT1. Apelin and exercise during pregnancy increase BAT markers, including UCP1, PRDM16, and PGC-1α, and increase the mRNA expression of Ucp1, Ppargc1a, Prdm16, cidea, Elovl3, and Cox7a1. This process increases body metabolism. In rodents, sympathetic stimulation by exercise activates β-adrenergic receptor 3 (β3-AR) and then stimulates BAT activity and WAT browning

Apelin stimulates the apelin receptor (APJ) to activate Gαi and Gαq and increases muscle metabolism. Calcium/calmodulin-dependent protein kinase 2 (CaMKK2) acts as a downstream molecule of Gαq for thermogenesis and is activated by exercise or apelin administration, while this is inactivated in the fetal muscle of obese mothers [17] (Fig. 1). The calcium gradient in myocytes is in part generated by the activity of SERCA, which transports Ca^2+^ into the SR [18, 19] (Fig. 1). Apelin activates AMPK through Gαq [20, 21] to stimulate glucose transporter 4 (GLUT4, in fat and muscle) and GLUT1 (in the heart and skeletal muscle) [22–24] (Fig. 1). Regular exercise activates AMPK to improve mitochondrial homeostasis, muscle metabolic capacity, increase blood glucose and improve metabolic disorders, and reduce blood glucose, blood insulin levels and insulin resistance in obesity and type 2 diabetes [21]. Exercise (30 min per day) activates PGC-1 and stimulates mitochondrial biogenesis, increases slow twitch fibers, and, as a result, increases glucose uptake through GLUT4 in the muscle of pregnant women [25] (Fig. 1). Similarly***,*** HIIT (50–60% to 70–95% maximum speed for 8 weeks, 4 sessions per week) also increases in PI3K and AKT1 to stimulate GLUT4 [26] (Fig. 1).

Apelin plays an important role in placental trophoblast nutrient uptake and improves fetal glucose homeostasis in animals [20], likely by upregulating the expression of GLUT1 and GLUT3 through hypoxia-inducible factor-1 (HIF-1) in trophoblast cells [55]. Exercise (40–60% of heart rate reserve) increases hypoxia to activate GLUT1 and increase glucose transport [56] (Fig. 1). Exercise during pregnancy (40–65% of VO_2max_ for 3 weeks) activates AMPK and PGC-1α in fetal muscle to stimulate the expression of UCP3 and sarcolipin leading to the inhibition of SERCA and raising intracellular levels Ca^2+^ to activate CaMKK2 and increase heat production from muscle contraction (Fig. 1). The increases in UCP3 and sarcolipin are maintained in the muscles of children of athletic mothers (trans-generational effects). The administration of apelin during pregnancy mimics the effects of maternal exercise on the activation of AMPK, CaMKK2, UCP3, and sarcolipin and emphasizes the role of apelin/AMPK signaling in improving fetal muscle growth in animals [17, 21].

### Apelin as a placentokine and exerkine in adipose and muscle tissue

Exercise-induced apelin release increases acetyl-CoA carboxylase (ACC) phosphorylation by activation of AMPK, which stimulates fatty acid oxidation [57]. There are increases in GLUT-1 and ACC protein levels and reductions in p-AMPKα and p-ACC protein levels in macrosomic human fetuses caused by excessive synthesis of fatty acids in the placenta of women with gestational diabetes [58] (Fig. 1). Apelin supplementation during pregnancy in animals increases brown fat tissue (BAT) markers, including UCP1, PRDM16, and PGC-1α and increases mRNA expression of Ucp1, Ppargc1a, Prdm16, cidea, Elovl3, and Cox7a1 in the circulation of female and male fetuses. Administration of apelin to pregnant mice increases BAT, oxidative phosphorylation, and mitochondrial activity and inhibits lipid synthesis and differentiation of white fat cells in fetal BAT [20] (Fig. 1). These data suggest a major role of apelin in the beneficial effects of maternal exercise (40–65% of VO_2max_ for a week), fetal BAT development [20], and increased mitochondrial biogenesis and thermogenesis in fetal muscle in animals [17] (Fig. 1). Exercise in pregnant rats increases mitochondrial biogenesis during fetal muscle development that is associated with increased oxidative muscle fibers, improved endurance capacity, and fewer metabolic disorders in the muscles of their offspring [59]. Maternal exercise (45–65% VO_2max_ for 8 weeks) in animals stimulates apelin levels to increase BAT weight by 38% and decrease WAT weight by 37% [20, 60]. Sympathetic stimulation by external stimuli such as exercise and norepinephrine activate β-3 adrenergic receptors (β3-AR) in rodents. Exercise in rats (60% VO_2max_, 6–9 weeks) activates the central nervous system and releases norepinephrine, stimulates BAT activity, and WAT browning via β3-AR [61] (Fig. 1). Levels of post-exercise apelin in women correlate with lean mass, insulin resistance, and insulin secretion [62]. On the other hand, increases in apelin secretion in female rats varies with the intensity of aerobic exercise, and hepatic sensitivity to insulin improves after exercise (70–75% VO_2max_) [63, 64].

### Apelin as a placentokine and exerkine in preeclampsia

It is estimated that preeclampsia occurs in 3–7% of pregnancies and that it is associated with decreased plasma apelin concentrations [65]. Apelin plays an important role in the formation of the fetal cardiovascular system and in the early development of the placenta during middle or late pregnancy, and modulates fetal angiogenesis and energy homeostasis [66]. Apelin mRNA and protein levels are reduced in women with preeclampsia [67], and treatment with apelin improves the symptoms of preeclampsia symptoms, stimulates eNOS/NO signaling, and reduces oxidative stress levels [27].

Aerobic exercise (40–60% of VO_2max_ for 60–150 min per week) or moderate-intensity resistance exercise is recommended for pregnant women [15]. Maternal exercise in both humans and animals increases apelin secretion by the placenta, promotes angiogenesis, and improves placental nutrient delivery [30, 31]. Regular exercise (moderate-intensity cycle ergometry, 130–140 beats/min, at least 3 h/week) in humans is associated with higher serum PlGF and lower sFlt-1 and sEng levels in late pregnancy [68]. Regular physical activity during early pregnancy in humans reduces the risk of preeclampsia by 35%, while vigorous physical activity (metabolic equivalent activities ≥ 6) reduces the risk of preeclampsia by 54%, and brisk walking (average walking speed ≥ 3 mph) reduces the risk of preeclampsia by 30–33%. Stair climbing is also inversely associated with the risk of preeclampsia. Increases in physical activity a year before pregnancy also reduce the risk of preeclampsia [12].

Apelin and its receptors are present in the heart, kidneys, and placenta and have a role in cardiovascular disorders during pregnancy. The pathophysiology of preeclampsia involves maternal endothelial dysfunction, abnormal placental development, hypoxia, oxidative stress, inflammation, and vascular dysfunction. An injured placenta releases anti-angiogenic proteins such as soluble fms-like tyrosine kinase 1 (sFlt-1) and soluble endoglin (sEng) that decrease signaling by vascular endothelial growth factor (VEGF) and placental growth factor (PGF). In addition, sEng inhibits the binding of transforming growth factor β1 (TGFβ1) to endoglin and prevents the activation of endothelial nitric oxide synthase (eNOS) and vasodilation. Therefore, excessive levels of sFlt-1 and sEng impair pregnancy-induced adaptation of the placental vasculature and contribute to endothelial dysfunction in women with preeclampsia [4].

Administration of apelin reduces blood pressure and increases heart rate, ejection fraction, and baroreflex sensitivity in rats with preeclampsia, and improves proteinuria, oxidative stress markers (4-HNE and NOX-4) [28] while also increasing mean arterial blood pressure, total urine protein, serum urea, creatinine, interleukin-6, endothelin-1, and malondialdehyde (MDA) levels, improving renal structure and decreasing serum NO levels in rats [69]. Acute exercise (1 h at 20 m/min, 10% incline) in animals increases AMPRK to stimulate VEGF mRNA levels [70]. In humans, aerobic exercise (at 50–65% of the maximum heart rate and for 60 min, three times a week for 16 weeks) increases NO production and decreases ROS levels in the placental vasculature [71].

### Leptin and gestational diabetes

**Fig. 2:**
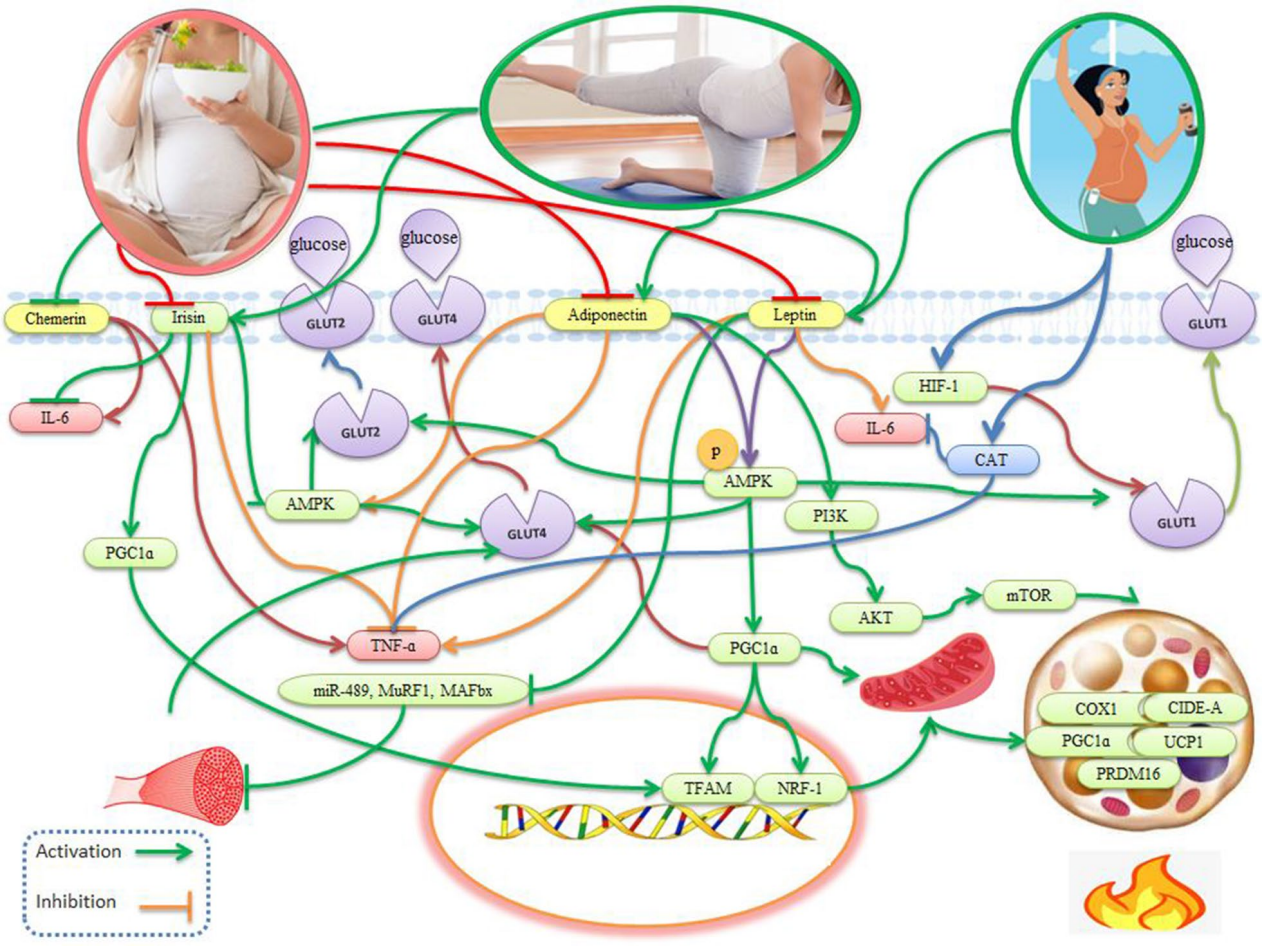
Effects of exerkines and placentokines on gestational diabetes in muscles and adipocytes. Leptin increases muscle mass by suppressing miR-489 and decreasing the expression of myostatin, MuRF1, and MAFbx. Then, by activating AMPK, leptin leads to increased hydrolysis and oxidation of fatty acids and muscle triglycerides (TG). The increase of leptin in pregnancy increases the production of TNF-α, IL-6, and IL-12 by monocytes. These inflammatory cytokines increase placental leptin mRNA expression and a vicious cycle is created to perpetuate the inflammatory state and exacerbate insulin resistance. Exercise during pregnancy suppresses TNF-ɑ and leptin and leads to a significant increase in leptin receptor expression and shows an increase in leptin sensitivity and a decrease in leptin resistance. Exercise acts as an antioxidant by increasing catalase (CAT) activity and antioxidant capacity. Adiponectin binding to its receptor activates AMPK, (p38-MAPK), (PPAR-α), PI3K, and Akt. Adiponectin improves insulin sensitivity and diabetes by increasing fatty acid oxidation and glucose absorption in skeletal muscles. Adiponectin negatively regulates inflammatory markers, such as TNF-α, IL-6, and CRP. A strong relationship between physical exercise and circulating adiponectin levels has been reported in healthy and diseased subjects. Exercise-induced release of irisin directly increases metabolism through activation of AMPK, PGC-1α, nuclear respiratory factor 1 (NRF1), and mitochondrial transcription factor A (TFAM), which increases GLUT4, mitochondria, and energy expenditure. By increasing the mRNA expression of UCP1 and cidea, irisin after exercise, causes the browning of white adipose tissue and increases thermogenesis. The expression of chemerin and inflammatory factors such as IL-6 and TNF-α are significantly upregulated in the peripheral blood of gestational diabetes patients. Exercise leads to a significant decrease in serum chemerin concentration and is associated with improved glucose absorption rate, reduced inflammation, and insulin sensitivity

Leptin is produced by the placenta and adipose tissue in humans. There are six leptin receptor isoforms (LepRa, LepRb, LepRc, LepRd, LepRe, and LepRf), with LepRb regulating the majority of leptin's functions, including inhibiting β-cell stimulation by blocking cAMP signaling [32, 35, 72]. Leptin regulates muscle glucose consumption by suppressing miR-489 (an inhibitor of muscle satellite cells), and increases muscle mass and muscle fiber size in mice by decreasing the expression of myostatin, muscle loop protein 1 (MuRF1), and muscle atrophy F-box (MAFbx) (Fig. 2), suggesting that treatment with leptin could increase muscle mass and muscle regeneration and repair [33]. Leptin activates AMPK to cause increased fatty acid hydrolysis and oxidation and generate muscle triglycerides in both humans and animals (TG) [34, 35].

Plasma levels of leptin in pregnant women are 2–3 times higher than in non-pregnant women, and increased leptin concentrations in early pregnancy predict preeclampsia and gestational diabetes [32, 73]. Leptin plasma levels increase by 150–200% in the second and third trimesters compared to the first trimester. The placenta, rather than adipose tissue, appears to be the primary site of leptin production, because increased leptin levels precede maternal weight gain. Maternal leptin levels decrease after delivery, and more than 90% of placental leptin is released into the maternal circulation. The human placenta also has elevated levels of leptin mRNA [32, 74]. Increased plasma leptin levels have often been associated with insulin resistance in human subjects, and a negative correlation between fasting plasma leptin and insulin sensitivity has been reported after adjusting for BMI. [75].

Thus, gestational diabetes is accompanied by increased expression of TNF-α and IL-6, with the exacerbation of an inflammatory state in the placenta. These inflammatory cytokines increase placental leptin mRNA expression to subsequently increase the production of TNF-α and IL-6 by monocytes [74] (Fig. 2). Thus, there is a vicious cycle created that perpetuates the inflammatory state and exacerbates insulin resistance, with a linear relationship between maternal hyperleptinemia concentrations and a ~ 5 times increased risk of gestational diabetes. Increased leptin levels also occur in the amniotic fluid, where an increase of 1 ng/ml in amniotic leptin levels increases the risk of developing diabetes by 4%. However, unchanged and decreased levels of leptin have been reported in patients with gestational diabetes [74]. Differences in disease severity or leptin measurement sites may partially explain these discrepancies [32].

### Exercise and leptin in gestational diabetes

Low physical fitness could contribute to gestational diabetes [75]. Exercise in humans and mice (moderate-intensity aerobic exercises, twice a week for 60–150 min a week) during pregnancy and in athletic mothers decreases fat (6%) and leptin levels (40%) [76]. Levels of TNF-ɑ and leptin during pregnancy in humans are lower in female athletes but return to normal levels with the cessation of exercise when there is an increase in fat mass [77]. Changes in TNF-α levels from pre-pregnancy to late pregnancy can predict insulin resistance in humans [78]. Exercise (30 min at 60–70% of maximum heart rate) during pregnancy increases antioxidant capacity but increases in catalase activity immediately after exercise in women with gestational diabetes are lower than in pregnant women without diabetes in humans [79]. Maternal exercise in mice (every day for 20 min, at a speed of 12.5 m per minute, 5 days a week, for 17 days) improves insulin sensitivity without changes in maternal weight or body composition and prevents excessive deposition of fat, hypoxia, and insulin resistance in the placenta and offspring [80]. Exercise in rats reduces glucose levels and prevents increases in leptin and, to some extent, triglyceride levels in male and female offspring [81].

Exercise (3 weeks of running) improves central leptin sensitivity and STAT3 activation and reduces plasma leptin levels in high-fat diet-fed rats during pregnancy and lactation [82]. Exercise during pregnancy ameliorates the harmful effects of a high-fat diet, such as increased lipid concentrations and plasma leptin and insulin levels in women. Maternal exercise in rats reduces leptin resistance by increasing the expression of Leprb, Stat3, Insr, Agrp, and Pomc in the fetal hypothalamus [83]. Running (8 m per minute for 30 min, 5 days per week) increases leptin receptor expression in rats' offspring [84]. Physical activity at 11, 24, and 36 weeks of pregnancy decreases triglyceride, TNF-α, and leptin levels in humans, with an inverse relationship between total activity (hours per week) or activity intensity and leptin levels in early pregnancy (< 16 weeks) [85]. Leptin levels are lower in women with high levels of activity and energy expenditure [86]. Hence, exercise during pregnancy can improve pregnancy outcomes in both low-risk and high-risk pregnancies in healthy women [87]. Future research should determine optimal exercise protocols to increase leptin sensitivity and reduce leptin resistance during pregnancy.

### Leptin and exercise in preeclampsia

Human preeclampsia is associated with insulin resistance and metabolic, immune, and angiogenic disorders [88]. Insulin resistance during pregnancy is related to gestational diabetes, hypertension, and preeclampsia in humans [89]. Maternal circulating leptin levels increase during pregnancy in both humans and rodents but decrease to pre-pregnancy levels at birth, indicating leptin production by the placenta. The human placenta synthesizes and releases leptin, and increased serum leptin levels occur in rats and humans with preeclampsia [90]. Levels of plasminogen activator inhibitor-1 (PAI-1) and inflammatory cytokines such as (TNF)-α and TNF receptor levels are increased in humans with preeclampsia [89], where there is a correlation between leptin serum concentrations and the inflammatory marker interferon-gamma-inducible protein 10 (IP-10). Raised serum leptin levels and sFlt-1/PlGF ratios increase the risk of preeclampsia, suggesting disturbances in angiogenesis [36], as supported by findings of increased levels of anti-angiogenic factors, such as sFLT1, sEndoglin, and endothelin 1. Therefore, it is not surprising that leptin has been measured in the amniotic fluid of women with preeclampsia [37]. Increases in leptin levels preceding preeclampsia (by ~ 2 months) suggest that leptin can be used as a biomarker of the risk of preeclampsia [75].

Leptin facilitates thrombus formation and atherosclerosis by stimulating the proliferation, migration, and calcification of vascular smooth muscle cells (VSMCs) [38]. In addition, hyperleptinemia in mice increases blood pressure, heart rate, pathological hypertrophy, left ventricular dysfunction, frequency of ischemic arrhythmias, systemic inflammation, and myocardial infarction [39]. Leptin stimulates the sympathetic nervous system, which acts to *reduce* leptin levels. Hence, long-term endurance exercise can reduce leptin levels by stimulating the sympathetic nervous system [91]. It has been reported that the amount of physical activity is inversely related to the risk of preeclampsia [92]. Average leptin levels are lower in women with greater physical activity (> 12.8 h per week) and energy consumption [93]. Active women have an approximately 50% reduced risk of gestational diabetes and a 40% reduced risk of preeclampsia compared to inactive women [94]. However, some studies have not observed significant differences in leptin levels between pregnant women with preeclampsia and control [88, 95]. Women with preeclampsia have higher minute ventilation, forced vital capacity, and a lower exercise tolerance [96]. Thus, it is likely that exercise can overcome these respiratory changes and increase the exercise tolerance of pregnant mothers, which is in keeping with the recommendation by the American College of Obstetricians and Gynecologists for exercise during pregnancy.

### Adiponectin and exercise and gestational diabetes

Adiponectin is secreted by the placenta and adipose tissue and has three receptors: adiponectin receptor 1 (AdipoR1), adiponectin receptor 2 (AdipoR2), and T-cadherin [15, 97]. Both AdipoR1 and AdipoR2 are abundant in skeletal muscle and liver, while T-cadherin is expressed on vascular endothelial cells and smooth muscles [98]. The binding of adiponectin to AdipoR1/R2 activates AMPK, p38 mitogen-activated protein kinase (p38-MAPK), peroxisome proliferator-activated receptor-α (PPAR-α), Ras-associated protein, Rab5, PI3K, and Akt [99] (Fig. 2). AMPK acts as an energy sensor that regulates cellular metabolism and is a potential target for the treatment of metabolic syndrome [98], making adiponectin important in glucose and lipid metabolism as low levels of adiponectin are associated with diabetes and cardiovascular disease [40].

Low levels of adiponectin in the first trimester of pregnancy are associated with increased insulin resistance and a higher risk of gestational diabetes in women [100]. Women with adiponectin concentrations less than 25% of baseline in the first trimester are 10 times more likely to develop gestational diabetes [101]. Obese women with gestational diabetes have lower serum adiponectin levels at 24–28 weeks of pregnancy [102]. The metabolic syndrome has a negative relationship between adiponectin and inflammatory markers, such as TNF-α, IL-6, and CRP**,** possibly due to the anti-inflammatory effects of adiponectin [98] (Fig. 2). Circulating adiponectin levels in animals and humans are negatively correlated with obesity [15]. Maternal intake of adiponectin in animals normalizes insulin sensitivity, insulin/mTORC1 signaling, nutrient transport, and fetal growth, where adiponectin supplementation in pregnant rats prevents the adverse effects of maternal obesity on placental function and fetal growth [103].

Adiponectin levels in humans can be increased by weight loss [40]. Adiponectin improves insulin sensitivity and diabetes by increasing fatty acid oxidation and glucose uptake in skeletal muscles and decreasing glucose production in the liver [41]. Adiponectin also increases insulin sensitivity in human pregnancy in women [99]. In addition, higher adiponectin levels are associated with greater physical activity, while prediabetes and diabetes are associated with decreased adiponectin levels. Restoring proper levels of adiponectin can be achieved by physical exercise, which also improves increased insulin sensitivity [104]. An acute bout of acute high- or low-intensity exercise (50–75% of VO_2_ peak for 1 week) increases serum adiponectin levels in humans [105]. Moderate-intensity interval training (MIIT) and high-intensity interval training (HIIT) both increase plasma adiponectin in obese adolescent females while decreasing the insulin resistance index [106]. Aerobic exercise by obese pregnant women increases adiponectin levels, while obese women without aerobic exercise have decreased levels of adiponectin.

Adiponectin levels decrease with higher activity in women with normal weight [107], which may be due to increased sensitivity of the ligand for its receptors and increased receptor numbers. In addition, exercise for 4 weeks increases circulating adiponectin levels and AdipoR1/R2 mRNA expression in the muscles of both males and females. Exercising for 3 h increases the expression of AdipoR1/R2 mRNA as well as the phosphorylation of AMPK and acetyl coenzyme A carboxylase in muscles without affecting circulating adiponectin levels, which could be due to low exercise levels [102].

### Adiponectin and exercise in preeclampsia

The number of women of reproductive age with hypertension (29.7%) and preeclampsia (32.1%) [15] is increasing. The relationship between adiponectin and preeclampsia is unclear, as some studies report no difference in adiponectin levels between pregnant women with preeclampsia and healthy pregnant women, suggesting that maternal serum adiponectin levels may be unrelated to preeclampsia [88]. Other studies report that preeclampsia and fetal growth restriction occur with changes in adiponectin levels [104]. The vascular protective effects of adiponectin are due to the reduction of expression of cell adhesion molecules, such as E-selectin, intercellular adhesion molecule (ICAM-1), vascular cell adhesion molecule (VCAM-1), and suppression of vascular inflammation, so that a reduction of adiponectin can cause vascular endothelial dysfunction [44].

High, medium, and low molecular weight adiponectin isoforms exert different functions. High molecular weight adiponectin protects endothelial cells from apoptosis, and the concentration of high molecular weight adiponectin is higher in women with preeclampsia. In contrast, the concentrations of medium and low molecular weight adiponectin do not change with preeclampsia [103]. The ratio of high molecular weight adiponectin to total adiponectin is also higher in preeclamptic women, suggesting a physiological feedback response to minimize endothelial damage in women with preeclampsia [42, 43]. Women with severe preeclampsia have higher plasma adiponectin concentrations than normal pregnant women, which may represent a compensatory feedback mechanism to counter the anti-angiogenic and pro-atherogenic effects of severe preeclampsia. Increased adiponectin levels in women with preeclampsia may be due to adiponectin resistance, decreased inflammation, or the severity of preeclampsia [44].

Women with severe preeclampsia and obesity (BMI ≥ 25 kg/m^2^) decrease adiponectin levels, while normal-weight women with preeclampsia increase adiponectin levels [108]. The mechanism of decreased adiponectin production appears to be a TNF-α-dependent pathway from visceral adipose tissue, as TNF-α acts as a potent inhibitor of adiponectin promoter activity. Hence, in obese women with preeclampsia, the concentration of adiponectin decreases, and the concentrations of TNF-α, CRP, and IL-6 plasma increase. Therefore, the reduction of adiponectin levels may be due to the stimulation of vascular inflammation in women with preeclampsia [44]. Some studies report that exercise during pregnancy can benefit both mother and child by affecting the placenta, possibly through angiogenic pathways [109]. Exercise-induced changes in adiponectin levels depend on several factors, such as pathological condition, type of exercise (endurance vs. resistance exercise), intensity (low, moderate, and intense), duration of exercise (acute vs. chronic, short vs. long), and gender [110]. Data on changes in adiponectin levels during preeclampsia and the effects of exercise during pregnancy have not been reported.

### Irisin as a placentokine and exerkine during gestational diabetes

Irisin has been proposed as a new target in the management of obesity and diabetes, because increases in irisin levels reduce insulin resistance [49]. Decreased plasma levels of irisin in the first trimester are associated with an increased risk of gestational diabetes and may be useful in the early identification of women at risk of gestational diabetes [45]. Levels of HbA1c, fasting blood glucose, 1-h glucose, 2-h glucose, and fasting insulin levels are higher in patients with gestational diabetes, while irisin levels are lower than in the control group, although umbilical cord blood irisin levels are unchanged [111, 112]. Serum irisin levels positively correlate with insulin resistance during the first trimester of pregnancy, with irisin levels being lower in the third trimester [113]. Circulating irisin levels are increased in women with postpartum gestational diabetes, which appears to be a compensatory feedback mechanism to modulate glucose homeostasis [114]. Serum irisin levels are higher in women with obesity and gestational diabetes compared to pregnant and non-obese women, which may be due to compensatory increases to regulate inflammatory factors and glucose homeostasis in obese people [46].

Levels of irisin in the cerebrospinal fluid positively correlate with serum irisin levels in non-obese and obese pregnant women, suggesting that irisin may have both central and peripheral roles in metabolism [46]. Levels of irisin are lower in less active pregnant women, while slight increases in active women (aerobic cycling exercise at least three times a week for 8 weeks) correlate negatively with fasting glucose, insulin concentration, and glycosylated hemoglobin, suggesting that exercise-induced irisin release during pregnancy could improve markers of glucose homeostasis in women and compensate for pregnancy-induced metabolic disturbances [115].

Exercise-induced irisin release can modulate muscle metabolism through AMPK activation [116]***.*** Treatment of animals with irisin increases levels of PGC-1α, nuclear respiratory factor 1 (NRF1), and mitochondrial transcription factor A (TFAM), leading to increased mitochondrial content and oxygen consumption [47, 48] (Fig. 2). Exercise (85% of the maximal heart rate) also increases PPAR-γ co-activator-1α (PGC-1α) levels, which in turn upregulates irisin levels. Exercise in humans and animals stimulates irisin levels, causing increased mRNA expression of UCP1, leading to the browning of subcutaneous and visceral adipose tissue and increasing thermogenesis [49]. Increased irisin levels occur in pregnant women after exercise, which can be used to promote exercise programs for pregnant women [117].

### Irisin as a placentokine in preeclampsia

Serum levels of irisin do not change during the first and second trimesters in preeclamptic women but are reduced in the third trimester [50, 113]. However, after 20 weeks of gestation in humans, there were no differences in irisin serum levels in patients with severe preeclampsia, patients with mild preeclampsia, and the control group [118]. Serum irisin negatively correlates with systolic and diastolic blood pressure in patients with preeclampsia [118, 119]. In addition, patients with preeclampsia have decreased serum irisin levels that are unrelated to body mass index and gestational age [120].

Maternal irisin levels in patients with mild preeclampsia are lower after cesarean delivery compared to vaginal delivery [121]. Plasma irisin level in singleton babies negatively correlates with maternal preeclampsia and positively correlates with gestational age and birth weight [119]. In addition, preeclampsia often involves ischemic injury and leads to increased placental cell death. Irisin has an anti-apoptotic role in placentas in the first trimester of women with preeclamptic women, where irisin prevents cell death by decreasing pro-apoptotic signaling cascades such as reducing PARP cleavage to induce DNA repair pathways and decreasing caspase-3 activity. Irisin increases the level of anti-apoptotic BCL2 compared to pro-apoptotic BAX and decreases the level of ROS. Irisin acts through the Akt signaling pathway to prevent apoptosis and increase cell survival [50].

Preeclampsia caused by placental hypoxia is due to endothelial dysfunction, which can be attenuated by the antioxidant and anti-inflammatory effects of irisin, as shown in rats with preeclampsia, where irisin decreases systolic blood pressure, diastolic blood pressure, ET-1, IL-6, MDA while increasing superoxide dismutase (SOD) levels, placental growth factor (PGF), and NO [122]. Since irisin is both an exerkine and a placentokine, future research should focus on the role of exercise in regulating irisin levels in preeclampsia.

### Chemerin as a placentokine and exerkine during gestational diabetes

Chemerin regulates immune function, obesity, and metabolism through three receptors [chemokine-like receptor 1 (CMKLR1), G-protein-coupled receptor 1 (GPR1), C–C motif chemokine receptor-like 2 (CCRL2)] that are expressed in the hypothalamus, pituitary gland, testis, ovary, and placenta [123]. Organs of the pregnant mother, such as the liver, placenta, and adipose tissue, increase the concentration of chemerin, with the expression of chemerin mRNA abundant in the liver but relatively low in the placenta [52, 124]. Chemerin levels in umbilical cord blood, peripheral blood, adipose tissue, and placenta tissue are higher during gestational diabetes [125, 126]. Chemerin levels positively correlate with fasting blood sugar, 2-h postprandial glucose, fasting insulin, insulin resistance index, C-reactive protein, and insulin dose [126].

Chemerin regulates adipogenesis and fat metabolism, reduces serum insulin levels and tissue glucose uptake in obese and diabetic mice, and causes insulin resistance in human muscle cells [51]. Serum chemerin levels are higher in obese women with normal glucose and those with normal weight and gestational diabetes compared to women with normal weight and normal glucose tolerance. Chemerin levels positively correlate with BMI, triglyceride (TG) levels, and insulin resistance [51]. Chemerin mRNA expression is higher in subcutaneous and visceral adipose tissue compared to the placenta in women in general but increases in visceral adipose tissue of obese women with gestational diabetes compared to non-obese women with normal glucose levels. As a result, increases in chemerin levels in gestational diabetes can contribute to insulin resistance and low-grade inflammation in obese women with gestational diabetes [52].

The expression of chemerin, fatty acid binding protein 4 (FABP4), and the inflammatory factors IL-6 and TNF-α are increased in the peripheral blood of gestational diabetes patients [127]. Levels of chemerin also are positively correlated with birth weight in humans [128]. In contrast, exercise decreases serum concentrations of chemerin in humans, and leads to improved insulin sensitivity and inflammation [129]. Circulating chemerin levels are decreased after a 24-week exercise plus diet program in humans, with reductions in biopsy tissues from abdominal adipose [130].

### Chemerin as a placentokine and exerkine in preeclampsia

Preeclampsia is associated with increased cardiovascular risks for the mother and the baby [131]. Serum chemerin levels increase as pregnancy progresses [132], leading to cardiovascular risks (hypertension, preeclampsia, coronary artery disease, and atherosclerosis) [53]. In addition, serum levels of chemerin are positively correlated with blood pressure, creatinine, free fatty acids, cholesterol, triglyceride (TG), leptin, and C-reactive protein. Among these, TG and leptin are independent predictors of chemerin levels during pregnancy. The average concentration of chemerin 6 months after delivery is higher in patients with preeclampsia [131, 133].

The expression and release of chemerin increases in the placenta of women with preeclampsia and are positively correlated with the ratio of soluble Fms-like tyrosine kinase-1 (sFlt-1) to placental growth factor (PlGF), a commonly used biomarker of preeclampsia. The overexpression of chemerin placental trophoblasts from mice causes a preeclampsia-like syndrome, hypertension, proteinuria, decreased trophoblast invasion, a disordered labyrinth layer, increased sFlt-1 and inflammatory markers (NFkB, TNF-α, and IL-1β), leading to growth restriction and reduced fetal weight in rats [54]. Chemerin may be a novel biomarker of preeclampsia, and inhibition of the chemerin/CMKLR1 pathway may be a promising new therapeutic strategy for the treatment of preeclampsia. A 6-month strength and endurance training program reduces chemerin levels in the circulation of overweight or obese people [134], suggesting that it may be useful to examine the possibility that exercise could reduce chemerin levels in women with preeclampsia.

## Conclusions

**Fig. 3:**
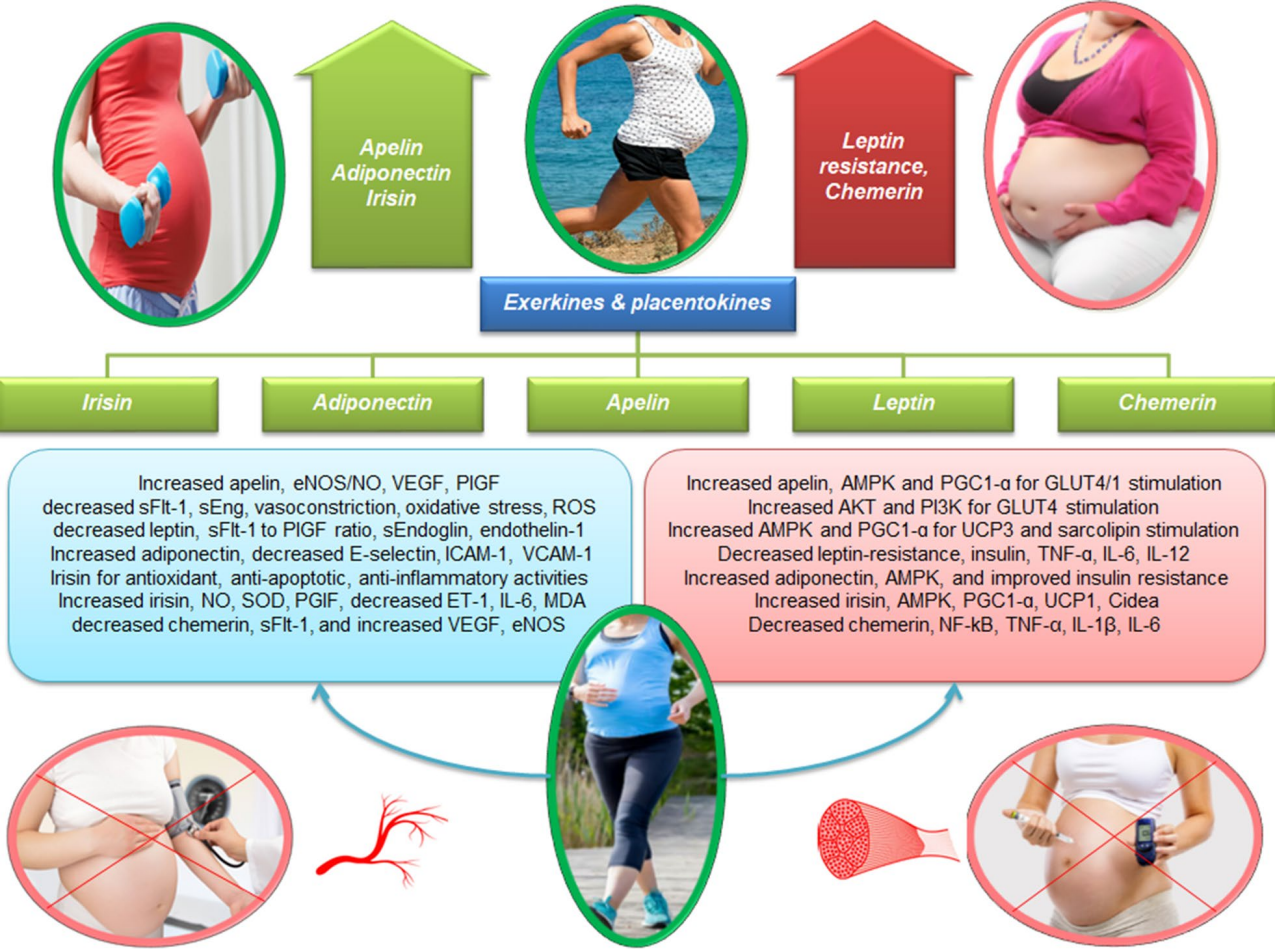
Effects of exerkines and placentokines on gestational diabetes and preeclampsia. Apelin and exercise increase AMPK, PI3k, and AKT1, leading to GLUT4, GLUT1, and PGC-1α stimulation in pregnant rats. This process then increases the expression of UCP3 and sarcolipin for thermogenesis. Acute exercise also increases VEGF mRNA via AMPK and, increases eNOS/NO production and decreases reactive oxygen species in placental vasculature. Decreased leptin induced by regular exercise during pregnancy is associated with increased serum concentration of PlGF and decreased sFlt-1, sEng, and endothelin-1. Exercise during pregnancy acts as an antioxidant and leads to a decrease in leptin, TNF-α, IL-6, and IL-12 levels. Exercise also improves central sensitivity to leptin, which is known as the phenomenon of leptin sensitization. Higher levels of adiponectin are associated with greater physical activity, and AMPK activation through adiponectin is an important process for glucose and lipid metabolism. Increasing the concentration of adiponectin leads to the decrease of E-selectin, ICAM-1, VCAM-1, and suppression of vascular inflammation. In active women, irisin is slightly increased and directly improves muscle metabolism through AMPK activation and then stimulates PGC-1α, which in turn leads to increased mRNA expression of UCP1 and cidea, increased thermogenesis, and increased adipose tissue browning. Irisin also leads to a significant decrease in systolic blood pressure, diastolic blood pressure, ET-1, IL-6, and MDA and a significant increase in SOD, PGF, and NO in patients with preeclampsia. In the peripheral blood of gestational diabetes patients, the expression of chemerin is positively correlated with the inflammatory factors IL-6 and TNF-α, while the level of chemerin decreases in the exercise + diet groups. Overexpression of chemerin through the CMKLR1 receptor in human trophoblasts leads to increased sFlt-1, decreased VEGF-A, and eNOS, which contributes to preeclampsia. On the other hand, 6 months of training reduces the level of chemerin significantly

Exercise can prevent gestational diabetes and preeclampsia by modulating levels of placentokines and exerkines, such as apelin, adiponectin, leptin, irisin, and chemerin (Table 1). Apelin and exercise increase AMPK, PGC-1α, PI3k, and AKT1 leading, to stimulation of GLUT4 and GLUT1 in pregnant rats while also stimulating eNOS/NO, increasing PlGF, decreasing sFlt-1, decreasing sEng, and reducing oxidative stress to improve preeclampsia symptoms (Fig. 3). Exercise (3 weeks of running) improves central sensitivity to leptin during pregnancy. Maternal exercise decreases the expression of markers of leptin resistance, such as Leprb, Stat3, Insr, Agrp, and Pomc in the hypothalamus. There is an increase in the peripheral blood concentrations of leptin, plasminogen activator inhibitor-1 (PAI-1), sFlt-1 to PlGF ratio, TNF-α, and TNF receptors in patients with preeclampsia, and also increased levels of anti-angiogenic factors, such as sFLT1, sEndoglin, endothelin 1, and leptin in the amniotic fluid of women with preeclampsia (Fig. 3).

Exercise increases adiponectin levels that regulate insulin sensitivity, decreases insulin resistance, and activates AMPK to regulate glucose and lipid metabolism. Low levels of adiponectin in the first trimester of pregnancy are associated with increased insulin resistance and a higher risk of gestational diabetes. Release of adiponectin by exercise stimulates AdipoR1 and AdipoR2 to reduce insulin resistance and mitigate metabolic syndrome. Increases in in adiponectin levels in preeclampsia have anti-inflammatory, and vascular protective activities and lead to decreased expression of cell adhesion molecules (E-selectin, ICAM-1, VCAM-1), and suppression of vascular inflammation (Fig. 3). Increased adiponectin levels may be a compensatory feedback mechanism to counter the anti-angiogenic, and pro-atherogenic effects of severe preeclampsia, adiponectin resistance, and reduced inflammation.

Inactive pregnant women have decreased levels of irisin, while increases in irisin in active women negatively correlate with fasting glucose, insulin concentration, and glycosylated hemoglobin. Irisin increases the mRNA expression of UCP1 and cidea, causing the browning of adipose tissue, increasing thermogenesis, and increasing energy consumption (Fig. 3). Irisin also decreases systolic and diastolic blood pressures, ET-1, IL-6, and MDA and increases SOD, PGF, and NO levels in mothers with preeclampsia in the third trimester (Fig. 3).

Expression levels of chemerin, IL-6, and TNF-α are increased in the peripheral blood of patients with gestational diabetes. Circulating chemerin levels are decreased by exercise and diet, and aerobic exercise and diet (exclusively and in combination) reduces inflammation and insulin resistance (Fig. 3). Overexpression of chemerin in human trophoblasts increases sFlt-1, decreases VEGF-A, and decreases eNOS levels, disrupts normal placental development through the CMKLR1 receptor, and causes fetal growth restriction and preeclampsia (Fig. 3). Strength and endurance training reduces chemerin levels in the circulation of overweight or obese people, but the effects of exercise on chemerin levels in women with preeclampsia are unclear.

## Data Availability

Not applicable.

## Abbreviations

ACC: Acetyl-CoA carboxylase
AdipoR1: Adiponectin receptor 1
AdipoR2: Adiponectin receptor 2
BAT: Brown fat tissue
β3-AR: β-Adrenergic receptor 3
CCRL2: C–C motif chemokine receptor-like 2
cidea: Cell death-inducing DNA fragmentation factor-like effector A
CMKLR1: Chemokine-like receptor 1
eNOS: Endothelial nitric oxide synthase
FABP4: Fatty acid binding protein 4
FNDC5: Fibronectin type III domain-containing protein 5
GPR1: G-protein-coupled receptor 1
GLUT4: Glucose transporter 4
HbA1c: Hemoglobin A1c
HIF-1: Hypoxia-inducible factor-1
HIIT: High-intensity interval training
HUVECs: Human umbilical vein endothelial cells
ICAM-1: Intercellular adhesion molecule
(IP)-10: Interferon-gamma-inducible protein
MAFbx: Muscle atrophy F-box
MAPK: P38 Mitogen-activated protein kinase
MDA: Malondialdehyde
MIIT: Moderate-intensity interval training
MuRF1: Muscle loop protein 1
NRF1: Nuclear respiratory factor 1
PAI-1: Plasminogen activator inhibitor-1
PGC-1α: PPAR-γ co-activator-1α
PIGF: Placental growth factor
PLP: Phospholamban
PPAR-α: Peroxisome proliferator-activated receptor-α
sEng: Soluble endoglin
sFlt-1: Fms-like tyrosine kinase 1
SLN: Sarcolipin
SOD: Superoxide dismutase
SR: Sarcoplasmic reticulum
TFAM: Mitochondrial transcription factor A
TG: Triglycerides
TGFβ1: Transforming growth factor β1
TNF-ɑ: Tumor necrosis factor α
VCAM-1: Vascular cell adhesion molecule
VEGF: Vascular endothelial growth factor
